# MsrB2 deficiency amplifies ECM-driven cardiac fibrosis under hypertensive stress

**DOI:** 10.3389/fphys.2026.1772933

**Published:** 2026-03-12

**Authors:** Ji Ho Yun, Suyeon Cho, Jong Youl Lee, Suji Kim, Seung Hee Lee

**Affiliations:** 1 Division of Endocrine and Kidney Disease Research, Department for Chronic Disease Convergence Research, Korea National Institute of Health (KNIH), Cheongju, Republic of Korea; 2 Division of Cardiovascular Disease Research, Department for Chronic Disease Convergence Research, Korea National Institute of Health (KNIH), Cheongju, Republic of Korea

**Keywords:** MsrB2, hypertensive cardiac hypertrophy, extracellular matrix remodeling, angiotensin II, oxidative stress, non-obese diabetes

## Abstract

**Background:**

Methionine sulfoxide reductase B2 (MsrB2), a mitochondrial redox enzyme essential for maintaining protein integrity under oxidative stress, has been implicated in diabetic cardiac remodeling. However, its contribution to hypertension-induced fibrosis remains unclear. Hypertension frequently coexists with diabetes and accelerates cardiac fibrotic remodeling, particularly in non-obese diabetic patients who may exhibit distinct metabolic and oxidative responses.

**Methods:**

We investigated the role of MsrB2 in extracellular matrix (ECM)-driven cardiac fibrosis using both animal and human hypertensive heart samples. MsrB2 expression was evaluated in non-obese (Goto-Kakizaki, GOTO) and obese (OLETF) diabetic rat models and in angiotensin II (Ang II)–infused MsrB2 knockout (KO) mice. Histological, biochemical, and transcriptomic analyses were performed to assess myocardial fibrosis, fibrosis-related signaling, and redox gene expression.

**Results:**

MsrB2 expression was markedly reduced in human hypertensive hearts and in the myocardium of non-obese diabetic rats, whereas it remained unchanged in obese diabetes despite similar increases in blood pressure. In MsrB2 KO mice, Ang II infusion provoked extensive interstitial and perivascular collagen deposition, accompanied by enhanced SMAD2/3 activation and upregulation of profibrotic ECM genes including *Col1a1*, *Col3a1*, *COMP*, and *LOX*. Transcriptomic profiling revealed strong enrichment of extracellular matrix and collagen-related pathways, along with increased expression of oxidative/inflammatory mediators such as *Spp1* and *Ccr2*, while antioxidant and mitochondrial quality-control genes (*Sdhaf2*, *Rnls*, *Mapk8*) were suppressed. These results indicate that MsrB2 deficiency shifts the myocardium toward a pro-oxidant and pro-fibrotic phenotype under hypertensive stress.

**Conclusion:**

Loss of MsrB2 amplifies ECM-driven cardiac fibrosis during hypertensive stress by promoting oxidative imbalance and SMAD2/3 activation. In non-obese diabetes, the concomitant reduction of MsrB2 expression may further accelerate hypertensive remodeling, highlighting a mechanism that could explain the higher incidence of cardiovascular complications observed in non-obese diabetic individuals. These findings identify MsrB2 as a critical redox regulator that restrains ECM-driven fibrosis and suggest that enhancing its activity could represent a therapeutic approach to prevent metabolic and hypertensive cardiac disease.

## 1 Introduction

Diabetes mellitus and hypertension are among the most common diseases and cardiovascular risk factors, and the pathogenic relationship between diabetes mellitus and hypertension is bidirectional (Triposkiadis et al., 2021; Tsimihodimos et al., 2018). In the United States, hypertension is highly prevalent among individuals with diabetes. Epidemiological evidence, including reports from the American Heart Association, indicates that hypertension occurs in approximately 50–80% of adults with type 2 diabetes, highlighting the substantial overlap between these two cardiometabolic conditions (Jia and Sowers 2021; Wei et al. 2011). Similarly, according to the Diabetes Fact Sheet in Korea 2024, comorbid hypertension is present in approximately 59% of Korean adults aged ≥30 years with diabetes, and the prevalence increases to nearly 70% among those aged ≥65 years. The coexistence of diabetes and hypertension markedly elevates cardiovascular risk and contributes to myocardial hypertrophy, interstitial fibrosis, and progression to heart failure, thereby amplifying the overall burden of cardiovascular disease.

Cardiac fibrosis, a hallmark of adverse cardiac remodeling, is characterized by excessive accumulation and cross-linking of extracellular matrix (ECM) proteins, leading to myocardial stiffening and dysfunction (Frangogiannis, 2021). Angiotensin II (AngII), a key effector of the renin-angiotensin system, plays a central role in promoting fibrosis by stimulating fibroblast proliferation and collagen deposition (Frangogiannis, 2021; Wang et al., 2015). In parallel, alterations in calcium-handling proteins, such as sarcoplasmic/endoplasmic reticulum Ca^2+^-ATPase 2a (SERCA2a), contribute to impaired cardiac relaxation and progression to heart failure (Tan et al., 2020). Methionine sulfoxide reductases (Msrs), particularly mitochondrial MsrB2, reverse methionine oxidation and preserve protein function under oxidative stress (Kim, 2013; Boschi-Muller and Branlant, 2014; Drazic and Winter 2014; Kim et al., 2014; Moskovitz and Smith, 2021; Reyes et al., 2018; Yin et al., 2012).

MsrB2 deficiency has been shown to exacerbate diabetic cardiomyopathy by impairing mitochondrial quality control, promoting fibrosis, and disrupting contractility (Lee et al., 2024; Lu et al., 2024). In our previous study, whole-body MsrB2 KO developed a metabolic and cardiovascular phenotype resembling non-obese diabetes, characterized by aggravated cardiac fibrosis and contractile dysfunction. This prompted us to investigate whether reduced MsrB2 expression might contribute to the exacerbation of hypertension, a comorbidity of diabetes, in this metabolic context. To elucidate this possibility, we first examined the functional role of MsrB2 using MsrB2 KO mice subjected to AngII-induced hypertension. The AngII model allowed us to dissociate hypertensive stress from broader metabolic dysfunction and directly test the causal role of MsrB2 in cardiac fibrosis. Furthermore, given that suppression of MsrB2 expression is associated with a non-obese diabetic phenotype, we sought to determine whether reduced MsrB2 expression in non-obese diabetes would exacerbate hypertension and accelerate fibrotic remodeling.

## 2 Result

### 2.1 Overview of experimental design

To provide a clear overview of the study design, the experiments were conducted in three sequential stages, each addressing a specific research question corresponding to the main figures.

In the first stage, we investigated whether MsrB2 deficiency exacerbates hypertension-induced cardiac fibrosis. Ang II–infused MsrB2 knockout (KO) mice were compared with wild-type (WT) controls, and fibrosis-related markers (Figures 2–4). MsrB2 deficiency markedly increased cardiac fibrosis and induced Smad2/3 activation. Collectively, these results outline a stepwise approach demonstrating that MsrB2 downregulation promotes hypertension-induced myocardial fibrosis.

Finally, we compared non-obese (GOTO) and obese (OLETF) diabetic rat models with their respective controls to examine whether different diabetes types influence blood pressure and cardiac MsrB2 expression (Figure 5). Both models exhibited elevated blood pressure, but only non-obese diabetes showed a marked reduction in cardiac MsrB2 expression, which was associated with more pronounced hypertension. These findings are consistent with our previous report demonstrating that MsrB2 deficiency aggravates diabetic cardiac complications.

### 2.2 MsrB2 decreased in human hypertensive hearts

**FIGURE 1:**
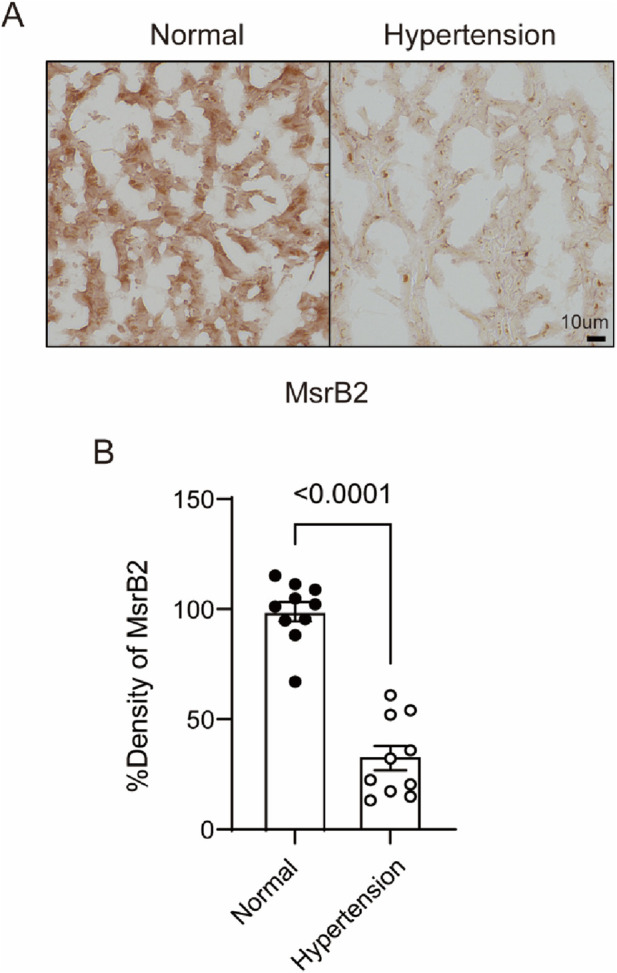
Decreased MsrB2 expression in hypertensive human heart tissue. **(A)** Immunohistochemical staining of MsrB2 in histological sections from normal and hypertensive human hearts. **(B)** Quantification of MsrB2 immunostaining intensity in histological sections. Data are presented as mean ± SD. Statistical significance was determined by an unpaired two-tailed *t*-test.

Our previous reports confirmed that MsrB2 regulates cardiac fibrosis under diabetes (Lee et al., 2024). This study utilized a hypertension-induced cardiac fibrosis model to verify the potential of MsrB2 as a therapeutic target. Hypertensive patients’ heart tissue exhibited decreased MsrB2 expression compared to that of normal subjects (Figure 1). Additional studies are needed to confirm that cardiac fibrosis induced by chronic diseases can be regulated by either increased or decreased MsrB2.

### 2.3 Cardiac fibrosis induced by MsrB2 depletion in hypertensive heart disease

**FIGURE 2:**
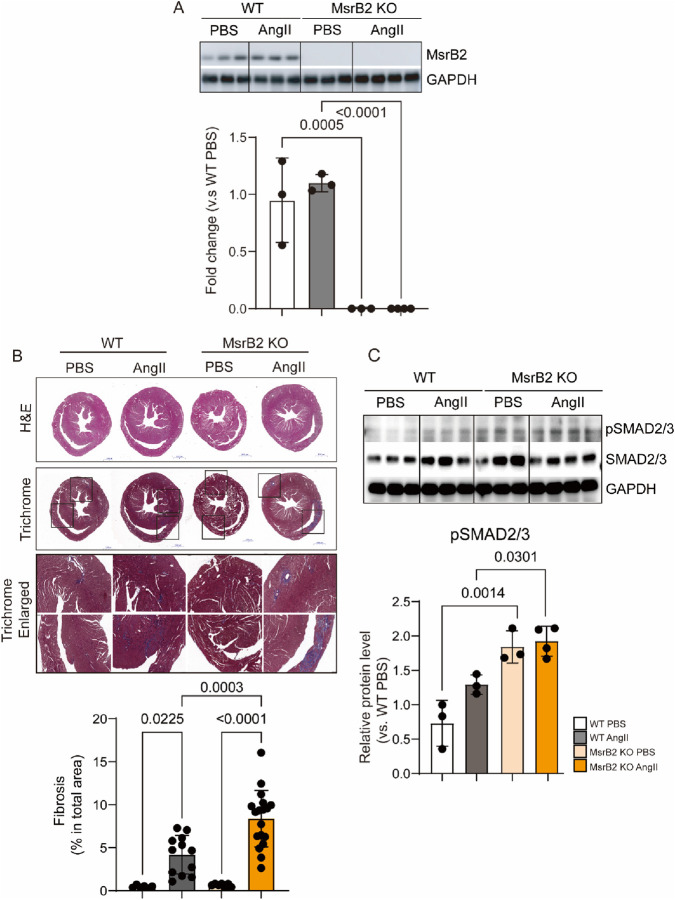
Cardiac fibrosis is induced by MsrB2 depletion in hypertensive mouse hearts. **(A)** Western blot analysis of MsrB2 protein expression in the same groups (n = 3–4 per group), with GAPDH as a loading control. Band intensities were normalized to GAPDH. **(B)** Representative hematoxylin and eosin **(H,E)** and Masson’s trichrome staining of heart sections from each group. Fibrotic areas were quantified using ImageJ (FIJI, version 1.53t, Wayne Rasband and contributors, National Institutes of Health, United States; https://imagej.nih.gov/ij/) software and expressed as a percentage of total tissue area. Black Boxed area was enlarged. **(C)** Western blot analysis of phosphorylated SMAD2/3 (pSMAD2/3), total SMAD2/3, and GAPDH protein expression (n = 3–4 per group). Band intensities were normalized to GAPDH. Representative cropped blots are shown; original blots with membrane are provided in Supplementary Information. Details of membrane cutting are described in the Methods. Data are presented as mean ± SD. Statistical significance was determined by one-way ANOVA.

Hypertensive cardiac disease was induced in wild-type (WT) and MsrB2 KO using an AngII infusion for 2 weeks (Figure 2). MsrB2 expression was reduced in the MsrB2 KO heart compared with WT group (Figure 2A). Cardiac fibrosis was substantially elevated in the MsrB2 KO AngII group, together with increased activation of the SMAD2/3 signaling pathway (Figures 2B,C).

**FIGURE 3:**
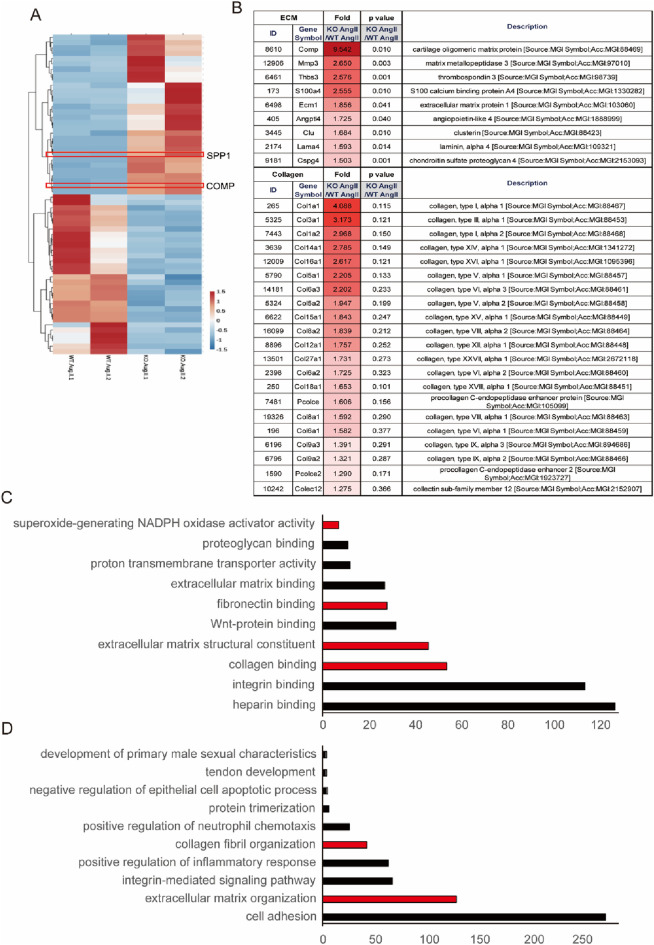
Activation of collagen and extracellular matrix (ECM)-related pathways in MsrB2 KO subjected to AngII infusion. **(A)** RNA-sequencing (RNA-seq) analysis of heart tissue from WT and MsrB2 KO, with or without AngII infusion. Differentially expressed gene (DEG) analysis was performed between WT AngII and MsrB2 KO AngII groups. **(B)** Heatmap showing genes significantly enriched in ECM- and collagen-related pathways. **(C)** Gene Ontology (GO) enrichment analysis highlights upregulated pathways related to extracellular matrix binding, fibronectin organization, and collagen fibril organization in MsrB2 KO Ang II hearts. **(D)** Kyoto Encyclopedia of Genes and Genomes (KEGG) pathway enrichment analysis showing activation of ECM-receptor interaction and collagen-associated pathways in MsrB2 KO AngII hearts.

**FIGURE 4:**
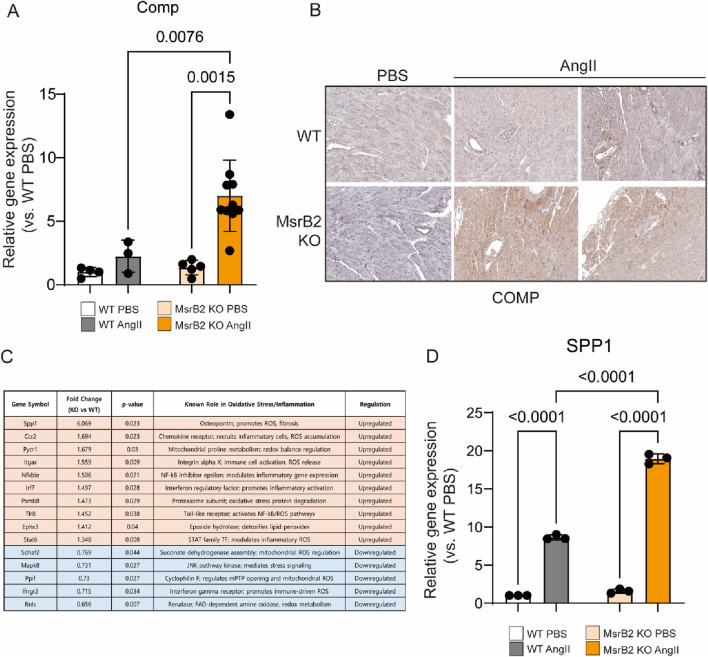
Increased collagen crosslinking associated with COMP and SPP1 in hearts of MsrB2 KO AngII mice. **(A,D)** Quantitative RT–PCR analysis of COMP transcript levels in mouse hearts (WT n = 4, MsrB2 KO n = 4, WT AngII n = 7, MsrB2 KO AngII n = 8), and Spp1 (WT n = 3, MsrB2 KO n = 3, WT AngII n = 3, MsrB2 KO AngII n = 3) **(B)** Immunohistochemical analysis of COMP expression in histological sections from WT and MsrB2 KO hearts with and without AngII infusion. **(C)** Differentially expressed genes related to inflammation in MsrB2 knockout (KO) *versus* wild-type (WT) hearts under AngII infusion. Fold change values represent KO/WT ratios. Genes in orange are upregulated; genes in blue are downregulated. Data are presented as mean ± SD. ; one-way ANOVA.

RNA sequencing revealed that MsrB2 KO infused with AngII showed upregulation of ECM- and collagen-related genes compared to WT AngII (Figures 3A,B **).** GO and KEGG analyses further indicated activation of pathways associated with extracellular matrix binding, fibronectin and collagen organization in MsrB2 KO AngII hearts (Figures 3C,D). qPCR analysis confirmed increased expression of COMP in MsrB2 KO AngII hearts (Figure 4A). COMP, previously reported to increase in heart failure and TAC-induced cardiac hypertrophy, plays a role in collagen organization and extracellular matrix stiffening. In the present study, COMP accumulated in perivascular areas with enhanced collagen deposition, and fibrosis was more pronounced in MsrB2 KO AngII hearts (Figure 4B). Consistent with the role of oxidative stress in hypertensive cardiac remodeling, MsrB2 deficiency was associated with increased expression of the ROS-related factor SPP1 (Figure 4C). Several genes associated with pro-oxidant and pro-inflammatory responses were markedly upregulated in MsrB2 KO hearts following AngII infusion, including *Spp1*, *Ccr2*, *Pycr1*, *Itgax*, *Nfkbie*, *Irf7*, *Psmb8*, *Tlr8*, *Ephx3*, and *Stat6*. In contrast, genes implicated in mitochondrial ROS regulation or antioxidant defense, such as *Sdhaf2*, *Mapk8*, *Ppif*, *Ifngr2*, and *Rnls*, exhibited reduced expression. These transcriptional changes indicate that the loss of MsrB2 reprograms the myocardium toward a pro-oxidant, pro-inflammatory phenotype under hypertensive stress (Figure 4C). Especially, highly elevated SPP1 in MsrB2 KO AngII group (Figure 4D).

### 2.4 Hypertension in diabetes

**FIGURE 5:**
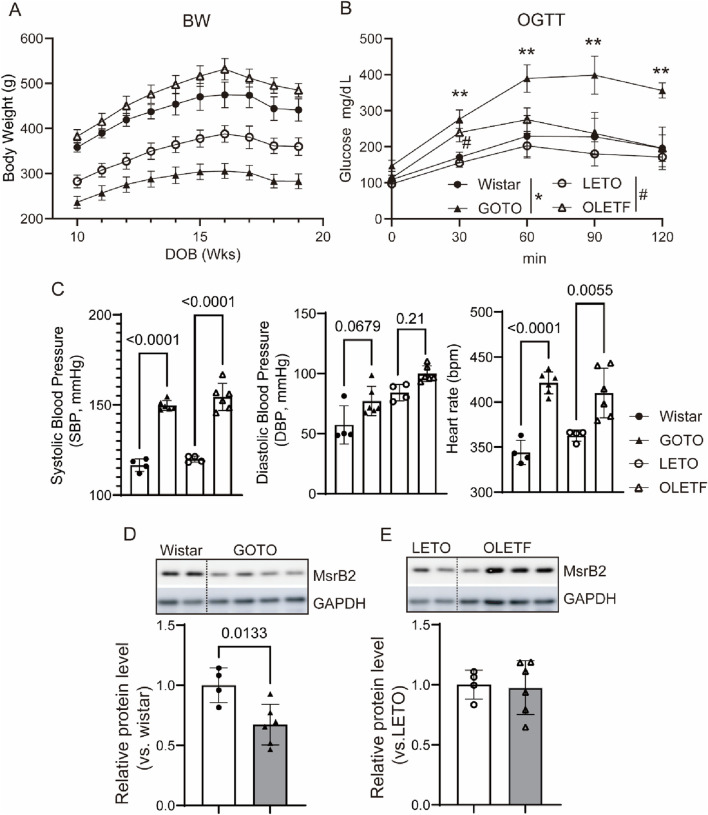
Hypertension in diabetic rat models and reduced MsrB2 expression. **(A, B)** Body weight and oral glucose tolerance test (OGTT) results in Goto–Kakizaki (GOTO) rats (n = 6) with control Wistar rats (n = 4), and in Otsuka Long-Evans Tokushima Fatty (OLETF) rats (n = 6) with control OLET rats (n = 4) at 18 weeks of age. **(C)** Systolic blood pressure, diastolic blood pressure, and heart rate in the same animal groups at 18 weeks of age. **(D,E)** Western blot analysis of MsrB2 protein expression in left ventricular tissue with GAPDH as a loading control. Band intensities were quantified and normalized to the GAPDH control. Representative cropped blots are shown; full-length blots with membrane edges are provided in Supplementary Information. Details of membrane cutting are described in the Methods. Data are presented as mean ± SD; one-way ANOVA.

To investigate the correlation between diabetes and hypertension, non-obese (GOTO) and obese (OLETF) diabetic rat models were used, with Wistar and LETO rats as respective controls (Bihoreau et al., 2017; Kawano et al., 1992) OGTT results showed impaired glucose tolerance in both GOTO and OLETF rats. GOTO rats exhibited a sustained increase compared to controls, while OLETF rats showed a transient elevation at 30 min (Figures 5A,B). At 10 weeks, both diabetic models showed elevated systolic and diastolic blood pressure without changes in heart rate. By 18 weeks, increases in blood pressure and heart rate were observed (Figure 5C). Expression of MsrB2, a critical factor in cardiac complications under diabetes, was reduced in the hearts of GOTO rats. At the same time, it remained unchanged in the hearts of OLETF rats compared to the control group (Figures 5D,E).

## 3 Discussion

This study demonstrates that MsrB2 is a critical redox regulator that protects the heart from hypertensive stress–induced fibrotic remodeling. Using complementary human, genetic, biochemical, and transcriptomic approaches, we show that reduced MsrB2 expression is a consistent feature of hypertensive hearts and that loss of MsrB2 markedly amplifies interstitial and perivascular fibrosis during angiotensin II (AngII)–induced hypertension. These findings extend previous reports implicating MsrB2 in diabetic cardiac injury and establish its broader relevance in hypertension-associated extracellular matrix (ECM) remodeling.

MsrB2 deficiency intensified fibrotic remodeling at both histological and molecular levels. Under AngII infusion, MsrB2 knockout (KO) hearts exhibited extensive collagen accumulation accompanied by strong activation of SMAD2/3, a canonical driver of TGF-β–mediated fibrosis. RNA-sequencing further revealed robust enrichment of ECM-related pathways, including collagen fibril organization, ECM–receptor interaction, and fibronectin assembly, indicating that loss of MsrB2 shifts the myocardial environment toward a matrix-producing phenotype. Among the differentially expressed genes, cartilage oligomeric matrix protein (COMP) emerged as a prominent fibrosis-associated molecule, showing increased transcript levels and localized protein accumulation in collagen-rich perivascular regions. Although COMP was not functionally interrogated in this study, its known role in stabilizing collagen networks suggests that elevated COMP may amplify ECM stiffness in MsrB2-deficient hearts.

Transcriptomic signatures also demonstrated that MsrB2 deficiency promotes a pro-oxidant and inflammatory state under hypertensive stress. Pro-inflammatory mediators (Spp1, Ccr2, Itgax, Irf7, Psmb8, Stat6) and oxidative stress–related genes (Pycr1, Ephx3) were strongly upregulated, while antioxidant or mitochondrial stress-adaptive genes (Sdhaf2, Mapk8, Ppif, Ifngr2, Rnls) were downregulated. This pattern indicates that MsrB2 loss not only disrupts redox homeostasis but may also impair mitochondrial stress responses. These alterations likely potentiate TGF-β/SMAD signaling and promote ECM gene expression, thereby synergistically accelerating fibrosis progression. Notably, Spp1 (osteopontin) was markedly increased in MsrB2 KO hearts, aligning with its established role as an oxidative/inflammatory amplifier in hypertensive and diabetic cardiac remodeling.

In addition to mechanistic insights from the animal model, our data from human hypertensive myocardium supports the clinical relevance of MsrB2 downregulation. The observation that MsrB2 expression is significantly reduced in hypertensive human hearts suggests that impaired MsrB2 activity may represent a common molecular feature of pathological pressure overload across species. Moreover, in non-obese diabetic rats—a metabolic condition associated with heightened cardiovascular risk—hypertension coincided with selective suppression of cardiac MsrB2 expression, whereas obese diabetes did not show such a reduction. These findings indicate that specific metabolic contexts, such as non-obese diabetes, may sensitize the heart to hypertensive remodeling by limiting MsrB2 expression.

Together, these results position MsrB2 as a molecular brake that constrains oxidative stress, inflammation, and ECM expansion in response to hypertensive stimuli. The protective role of MsrB2 is consistent with its known function in reversing methionine oxidation and maintaining mitochondrial protein quality. Although mitophagy flux was not directly assessed in this study, previous work has shown that MsrB2 regulates Parkin-mediated mitophagy, suggesting that impaired mitochondrial quality control may contribute to heightened vulnerability under AngII stress. Future studies incorporating functional assays of mitochondrial turnover will be important to clarify this mechanism.

This study has several limitations. First, blood pressure was assessed using noninvasive tail-cuff measurements, which may be less precise than telemetry-based monitoring. Second, COMP and SPP1 were identified as potential fibrotic mediators but were not mechanistically interrogated; therefore, their causal involvement remains to be validated. Third, MsrB2-dependent mitochondrial signaling was not directly evaluated, and conclusions regarding redox or mitophagy pathways should be interpreted within this context. Although mitochondrial functional assays were not directly performed, transcriptomic data indicated that antioxidant and mitochondrial stress-response genes were suppressed under MsrB2 deficiency. Future studies, including mitochondrial respiration and mitophagy assays, are warranted to further elucidate the mechanistic basis. Further validation of RNA-seq–identified candidate genes at the protein level will be important to confirm transcriptional changes and strengthen the mechanistic interpretation. Despite these limitations, the multi-layered evidence provided here, including human tissue analysis, genetic mouse models, histological quantification, and transcriptomic profiling, strongly supports a central role for MsrB2 in modulating hypertensive cardiac fibrosis.

Both non-obese and obese forms of diabetes are frequently accompanied by hypertension; however, in non-obese diabetes, this condition is associated with a marked reduction in cardiac MsrB2 expression. This concomitant suppression of MsrB2 may predispose the myocardium to greater fibrotic remodeling and oxidative injury, thereby increasing the risk of cardiovascular complications. These findings imply that individuals with non-obese diabetes—particularly those prevalent in Asian populations—may require earlier and more intensive clinical management to prevent hypertension-associated cardiac complications. From a public health perspective, this highlights an underrecognized vulnerability in non-obese diabetes and underscores the importance of proactive cardiovascular surveillance in this population.

In conclusion, our findings identify MsrB2 as an essential protective factor that restrains ECM-driven fibrotic remodeling during hypertensive stress. Enhancing MsrB2 activity in cardiomyocytes, either through gene-based approaches or pharmacological activation, may represent a promising therapeutic strategy for preventing hypertension- and diabetes-related cardiac remodeling. This work expands the pathophysiological significance of MsrB2 beyond diabetes and underscores its potential as a translational target in metabolic and hypertensive heart disease. As well, future investigations will aim to determine the mechanistic roles of key fibrosis mediators, including COMP and SPP1, in MsrB2-regulated cardiac remodeling.

## 4 Methods

### 4.1 Preparation of mice and a hypertension mouse model

Male Otsuka Long-Evans Tokushima Fatty (OLETF) and age-matched control Long-Evans Tokushima Otsuka (LETO) rats, as well as male Goto-Kakizaki (GOTO) and Wistar rats (four to five weeks old; Japan SLC, Shizuoka, Japan), were used. All animals were housed individually in polycarbonate cages under controlled temperature (22 °C ± 3 °C), humidity (55% ± 15%), and a 12-h light/dark cycle, with free access to standard rodent chow (Teklad 2918 °C, Inotiv, United States) and microfiltered tap water. Blood pressure and heart rate were measured using a noninvasive tail-cuff system (Kent Scientific, United States) after 5 days of acclimation to minimize stress. On the measurement day, animals were maintained at 37 °C to ensure stable tail blood flow, and readings were taken five times and averaged for analysis. For oral glucose tolerance testing, rats were fasted overnight and blood glucose levels were measured at 0, 30, 60, 90, and 120 min after oral glucose administration.

Wild-type (WT) and MsrB2 knockout (KO) mice were on a C57BL/6 J background. Four experimental groups were used: WT (PBS), WT (AngII), MsrB2 KO (PBS), and MsrB2 KO (AngII).

Each group included 5–7 mice (male, 10–12 weeks old). Hypertension was induced by continuous infusion of angiotensin II (AngII, 1.5 μg/kg/min) for 2 weeks using osmotic minipumps (Li et al., 2021). All surgical procedures were performed under deep anesthesia with ketamine (100 mg/kg, i.p.) and xylazine (Rompun, 10 mg/kg, i.p.), and tissues were collected under isoflurane (3%–5% in oxygen) anesthesia until complete respiratory arrest was achieved. This approach ensured humane euthanasia consistent with the AVMA Guidelines for the Euthanasia of Animals (2020).

All animal experiments were approved by the Institutional Animal Care and Use Committee of the Korea Disease Control and Prevention Agency (KDCA-IACUC-22-04) and performed at the Korea National Institute of Health (KCDC-11-2-26) facility, in accordance with national and institutional regulations. The study adhered to the ARRIVE guidelines (https://arriveguidelines.org).

Mice were randomly assigned to the experimental group. Group sizes were determined based on previous studies and pilot experiments to ensure adequate statistical power while minimizing the use of animals. No formal sample size calculation was performed, but biological replicates (n = 3–9 per group, as detailed in figure legends) were included. Investigators were blinded to genotype during histological and molecular analyses.

### 4.2 Immunohistochemistry and quantification

Slides of human heart tissue, both hypertensive and normal, were purchased from Novus(Catalog No. NBP-77723).The tissue sections underwent a series of treatments: deparaffinization and rehydration using a graded ethanol series and distilled water; incubation with a Proteinase K working solution for 20 min at 37 °C in a humidified chamber; and treatment with 0.3% H_2_O_2_ in DW for 30 min to block endogenous peroxidase activity. Following these treatments, the tissue sections were rinsed three times for 10 min in PBS and incubated with 3% TritonX-100 for 10 min for permeabilization. After washing, the samples were incubated with 10% normal horse serum for 1 h to block non-specific antibody binding. The tissue sections were then rinsed three times for 10 min in PBS and incubated with primary antibody against MsrB2 (Abcam, ab175845, 1:100). After another round of PBS washes, the sections were incubated with secondary antibodies and treated with ABC solution (Vectastain ABC kit, PK-4000) for 30 min. The sections were then stained with DAB according to the manufacturer’s protocol, dehydrated using a graded ethanol series and xylene, and photographed using EVOS™ M5000 (Thermo Fisher Scientific) at 10x, ×20, and ×40 magnification. To measure the optical density of MsrB2 immunoreactivity, 20x brightfield images were captured and imported into ImageJ software. The images were converted to black-and-white and inverted, and the mean value per pixel was measured within each region of interest. A simple random image captured up to 10 photos of tissue sections.

### 4.3 Western blotting

The whole hearts were immediately frozen in liquid nitrogen and ground into fine powder using a pre-chilled mortar and pestle. The powdered tissue was then lysed in ice-cold buffer containing 50 mM Tris-HCl (pH 7.4), 150 mM NaCl, 0.25% Triton X-100, and a complete protease and phosphatase inhibitor cocktail (Sigma-Aldrich). Protein concentration was determined using the bicinchoninic acid (BCA) assay (Thermo Fisher Scientific), and equal amounts of protein were loaded onto 8%–16% Criterion™ TGX™ Precast Midi Protein Gels (Bio-Rad) and electrophoresed at 100 V. Proteins were then transferred to PVDF membranes(Millipore, Billerica, MA) at 95 V for 1 h. The membrane was blocked with 5% non-fat dry milk and incubated with primary antibodies (MsrB2 homemade (Lee et al., 2024; Lee et al., 2019) 1:1000, pSMAD2/3 Cell Signaling #8828(Lot#7) 1:1000 and SMAD2/3 Cell Signaling #3102(Lot#9) 1:1000 overnight in a cold room. The membrane was incubated with a secondary antibody conjugated to horseradish peroxidase (HRP) (Gendepot) and developed with a chemiluminescent substrate (Santa Cruz and Thermo Fisher Scientific). The band images were obtained using the Luminescent Image Analyzer (ImageQuant LAS 4000 Mini, GE Healthcare Bio-Sciences AB). Band intensity was analyzed using ImageJ analysis software (ImageJ, version 1.46r, Wayne Rasband, National Institutes of Health, United States; https://imagej.nih.gov/ij/), and the signal intensity of each band was first normalized to GAPDH and then expressed as a fold change relative to the WT or non-treated group. In some experiments, the amount of biological sample was limited, making it impractical to run multiple full-size gels for each target protein. Each blot represents an independent biological sample from an individual animal. To allow targeted probing, membranes were cut prior to antibody incubation. All available original blot images with visible membrane edges are included in the Supplementary Information for transparency, and figure legends indicate where membranes were cropped.

### 4.4 Histological analysis of hearts

Two weeks after angiotensin II or PBS infusion, animals were sacrificed and hearts were arrested in end-diastole. The left ventricle was weighed, embedded in paraffin or glycol methacrylate, and sectioned. Hematoxylin-eosin staining was used to measure cardiomyocyte area, and Masson’s trichrome staining was used to assess fibrosis. The fibrotic area was calculated as the ratio of fibrotic to total tissue area. Microscopic analysis was performed using an Axiophot microscope and quantified with AnalysisSIS 3.2 (Soft-Imaging System, Germany) and ImageJ Fiji software (version 1.53t).

### 4.5 RNA-sequencing and bioinformatic analysis

Total RNA was isolated from the hearts of both WT and MsrB2 knockout mice heart (nonDM and DM). RNA sequencing was performed by Bioneer in Korea. The libraries were prepared for 150 bp paired-end sequencing using TruSeq Stranded Total RNA Sample Prep Kit with Ribo-Zero H/M/R. mRNA molecules were purified and fragmented from 2 μg of total RNA using oligo(dT) magnetic beads. The fragmented mRNAs were synthesized as single-stranded cDNAs through random hexamer priming. By using this as a template for second-strand synthesis, double-stranded cDNA was prepared. After a sequential process of end repair, A-tailing and adapter ligation, cDNA libraries were amplified with PCR (Polymerase Chain Reaction). The quality of these cDNA libraries was evaluated with the Agilent 2,100 BioAnalyzer (Agilent, CA, United States). They were quantified with the KAPA library quantification kit (Kapa Biosystems, MA, United States) according to the manufacturer’s library quantification protocol. Following cluster amplification of denatured templates, sequencing was progressed as paired-end (2 × 150 bp) using Illumina Novaseq 6,000 (Illumina, CA, United States). They were quantified using the KAPA Library Quantification Kit (Kapa Biosystems, MA, USA) according to the manufacturer’s protocol. Following cluster amplification of denatured templates, sequencing was performed as paired-end (2×150 bp) using an Illumina NovaSeq 6000 (Illumina, CA, USA). performed by Bioneer in South Korea.

Differential expression analysis was performed using standard bioinformatic pipelines. Gene Ontology (GO) and Kyoto Encyclopedia of Genes and Genomes (KEGG) analyses were conducted to identify enriched pathways. RNA-seq data have been deposited in a public repository (https://www.ebi.ac.uk/biostudies/arrayexpress/studies/E-MTAB-15275).

### 4.6 Quantitative real-time PCR (qPCR)

cDNA was synthesized using the Superscript IV Reverse Transcriptase (Thermo Fisher). qPCR was performed with Power SYBR™ Green PCR Master Mix (Thermo Fisher) on a SG/QuantStudio 6 Flex (Thermo Fisher). Gene expression was normalized to Gapdh using the ΔΔCt method.

### 4.7 Statistical analysis

All data are presented as mean ± standard deviation (SD) unless otherwise stated. Statistical comparisons between multiple groups were performed using one-way ANOVA followed by Tukey’s *post hoc* test, as outlined in the individual experiments. A p-value <0.05 was considered statistically significant. All analyses were conducted using GraphPad Prism (version 9.0).

## Data Availability

The RNA sequencing datasets generated and analyzed in this study have been deposited in the ArrayExpress database under accession number E-MTAB-15275, accessible at: https://www.ebi.ac.uk/biostudies/arrayexpress/studies/E-MTAB-15275).
